# Brief cardiovascular imaging with pocket-size ultrasound devices improves the accuracy of the initial assessment of suspected pulmonary embolism

**DOI:** 10.1007/s10554-018-1382-5

**Published:** 2018-05-30

**Authors:** Dominika Filipiak-Strzecka, Jarosław D. Kasprzak, Piotr Lipiec

**Affiliations:** Department of Cardiology, Bieganski Hospital, Medical University of Lodz, Kniaziewicza 1/5, 91-347 Lodz, Poland

**Keywords:** Pocket-size imaging devices, Pulmonary embolism, Compression ultrasound test, Wells rule, Revised Geneva score

## Abstract

Pulmonary embolism onset is frequently neglected due to the non-specific character of its symptoms. Pocket-size imaging devices (PSID) present an opportunity to implement imaging diagnostics into conventional physical examination. The aim of this study was to test the hypothesis that supplementation of the initial bedside assessment of patients with suspected pulmonary embolism (PE) with four-point compression venous ultrasonography (CUS) and right ventricular size assessment with the use of PSID equipped with dual probe could positively influence the accuracy of clinical predictions. A single-centre, prospective analysis was conducted on 100 patients (47 men, mean age 68 ± 13 years) with suspected PE. Clinical assessment on the basis of Wells and revised Geneva score and physical examination were supplemented with CUS and RV measurements by PSID. The mean time of PSID scanning was 4.9 ± 0.8 min and was universally accepted by the patients. Fifteen patients had deep venous thrombosis and RV enlargement was observed in 59 patients. PE was confirmed in 24 patients. If the both CUS was positive and RV enlarged, the specificity was 100% and sensitivity 54%, ROC AUC 0.771 [95% CI 0.68–0.85]. The Wells rule within our study population had the specificity of 86% and sensitivity of 67%, ROC AUC 0.776 (95% CI 0.681–0.853, p < 0.0001). Similar values calculated for the revised Geneva score were as follows: specificity 58% and sensitivity 63%, ROC AUC 0.664 (95% CI 0.563–0.756, p = 0.0104). Supplementing the revised Geneva score with additional criteria of CUS result and RV measurement resulted in significant improvement of diagnostic accuracy. The difference between ROC AUCs was 0.199 (95% Cl 0.0893–0.308, p = 0.0004). Similar modification of Wells score increased ROC AUC by 0.133 (95% CI 0.0443–0.223, p = 0.0034). Despite the well-acknowledged role of the PE clinical risk assessment scores the diagnostic process may benefit from the addition of basic bedside ultrasonographic techniques.

## Introduction

Pulmonary embolism (PE) is a common, oftentimes misdiagnosed emergency cardiovascular state burdened with potentially fatal consequences. Symptoms are non-specific varying from chest pain, shortness of breath, cough and hemoptysis to syncope and cardiac arrest [1]. Annually over 10 million patients in the United States seek medical help with the complaints of dyspnea, chest pain or both. It is estimated that over 600,000 cases of PE are diagnosed in United States every year [2]. Majority of death related cases of PE occur when the condition was not diagnosed in time [3].

According to current guidelines [1, 4–6], the first step in the diagnostic protocol of PE is determination of its clinical probability. For this purpose several scoring systems are suggested. The Wells rule and the modified Geneva score are among the most validated scales used for PE clinical risk assessment. In order to reduce the number of unnecessary procedures, guidelines recommend first to identify patients with a very low probability of the condition, so that referral for further diagnostics can safely be withheld. The remaining patients are categorized into one of the recommended diagnostic paths; which in most cases involves further blood testing or—perceived as a golden standard—Multi-Slice CT Angiography. Undoubtedly, unjustified referral for diagnostic procedures involving a radiographic exposure results in excessive cost of care, increased use of hospital resources, significant risk of contrast-associated acute renal failure and exposure-dependent malignancies [7]. On the contrary, in certain cases even with calculated low clinical risk (evaluated by established scoring systems) postponing of final diagnosis and late introduction of proper treatment may cause fatal consequences. For this reason a persistent search for new diagnostic modalities improving initial assessment of patient condition, which could increase the accuracy of grading clinical risk of PE is continued. However, a novel diagnostic protocol needs to meet certain requirements in order to gain clinical acceptance: it has to be widely available, quick to implement and should be cost effective. A method/strategy where all patients suspected of PE should undergo ultrasonographic examination as part of initial evaluation proves challenging in the urgency of the emergency department. Certain constraints such as urgent transportation to echocardiographic laboratory, use of high-end equipment and need for trained echocardiographer generate high costs. The trend to miniaturize echocardiographic devices has led to the creation of pocket-size imaging devices (PSID). With the ultraportability being their biggest advantage PSIDs may be used in almost every clinical setting, including the emergency room. Moreover, with an interface optimized for ease of operation, PSIDs present easy access to a wide range of users. It has been shown, that with targeted training basic information can be obtained with the use of PSIDs by non-echocardiographers [8–10]. We hypothesized, that in patients with suspected pulmonary embolism augmentation of initial emergency room assessment with four-point compression venous ultrasonography (CUS) and right ventricular size assessment with the use of PSID equipped with dual probe can improve the diagnostic accuracy of established clinical prediction rules.

## Materials and methods

This was a prospective study conducted between February 2015 and May 2016. 100 consecutive patients (47 males, mean age 68 ± 13 years) who were referred to our department during office hours (8 a.m.–4 p.m.) or during the 24-h medical shift of the resident performing examination (D.S.) were included in our analysis. The inclusion criterion was the suspicion of pulmonary embolism based on the medical history and basic physical examination only. All patients reported dyspnea as their main symptom, in some cases with concomitant chest pain (49%), cough (17%) or tachycardia (27%). If such a diagnosis was suggested, in all cases the investigator (D.S.) was informed and continued with further diagnostic procedures. All patients underwent clinical assessment in the emergency room on the basis of the Wells rule and the revised Geneva score, including the evaluation of medical history (previous episode of pulmonary embolism or deep vein thrombosis, recent surgery), current medical condition (active cancer, heart rate, clinical signs of DVT) and reported symptoms (hemoptysis) (Table 1). Subsequently, the regular physical examination was supplemented with short, focused bedside ultrasonographic assessment consisting of four-point compression venous ultrasonography and measurements of the right ventricle performed by cardiology resident with the use of PSID. The operator’s training in echocardiography was included as part of her residency program and was based on six-month rotation in the echo lab. It included conducting and analyzing transthoracic echocardiographic examinations under specialist supervision, as well as a basic vascular examinations such as compression ultrasound test. This study was conducted in accordance with the amended Declaration of Helsinki. Informed consent was obtained from individual participants included in the study. The study protocol was approved by bioethics committee of our institution (Decision No. RNN/8/10/KE with the Supplement No. KE/2011/15).

**Table 1:** The original and modified versions of Wells rule and revised Geneva score used in the study

Wells rule				
Original version	Modified version	Points	% of patients	
Previous PE or DVT	Previous PE or DVT	1.5	7	
HR > 100 b.p.m	HR > 100 b.p.m	1.5	27	
Surgery or immobilization within the past 4 weeks	Surgery or immobilization within the past 4 weeks	1.5	9	
Hemoptysis	Hemoptysis	1	0	
Active cancer	Active cancer	1	2	
Clinical signs of DVT	Positive CUS result	3/4	9	15
Alternative diagnosis less likely than PE	Alternative diagnosis less likely than PE	3	16	
–	RV enlargement Basal diameter (4CH) ≤ 47 mm Basal diameter (4CH) > 47 mm	1 2	59 35 19	

Revised Geneva score				
Original version	Modified version	Points	% of patients	
Previous PE or DVT	Previous PE or DVT	3	7	
HR 75–94 b.p.m ≥ 95 b.p.m	HR 75–94 b.p.m ≥ 95 b.p.m	3 5	15 27	
Surgery or fracture within the past month	Surgery or fracture within the past month	2	9	
Hemoptysis	Hemoptysis	2	0	
Active cancer	Active cancer	2	2	
Unilateral lower limb pain	Positive CUS result	3	10	15
Pain on lower limb deep venous palpation and unilateral oedema		4	6	
Age > 65 years	Age > 65 years	1	63	
–	RV enlargement Basal diameter (4CH) ≤ 47 mm Basal diameter (4CH) > 47 mm	1 2	59 35 19	

### Pocket-size imaging device

The pocket-size imaging device used in this study was V-Scan (GE Vingmed Ultrasound, Horten, Norway) equipped with dual probe, combining two transducers in one probe- the phased array (frequency range of 1.7–3.8 MHz, image sector limited to 75°, depth range 6–24 cm) and the linear probe (frequency range of 3.4–8.0 MHz, aperture width of 2.9 cm, maximum depth of 8 cm). The four-point compression ultrasound tests were performed using linear probe and the vascular preset, whereas the right ventricle assessment with the phased array probe and cardiac preset.

### Compression ultrasonography

**Fig. 1:**
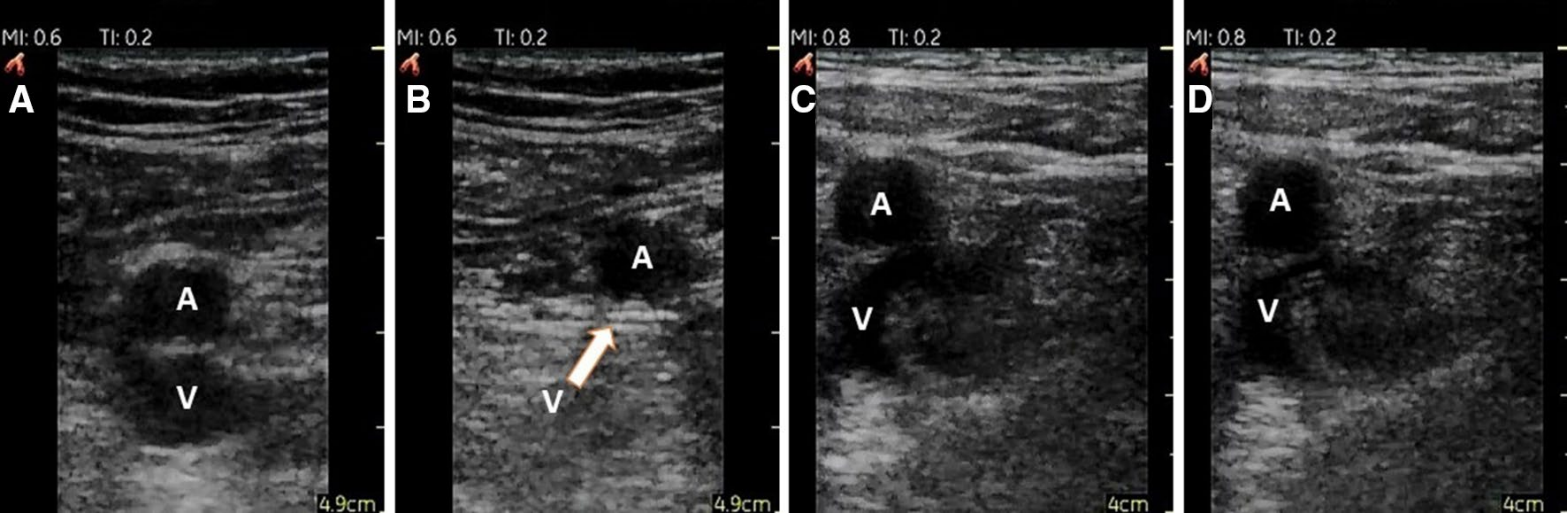
Compression ultrasound test of common femoral vein performed with the use of PSID in two patients: without thrombosis (panel **a, b**) and with venous thrombosis (panel **c, d**). Panel **a, c**—baseline; panel **b, d**—compression. Panel **b**—vein completely compressed; panel **d**—abnormal study indicating venous thrombosis; *A* femoral artery; *V* femoral vein

The examination was performed in the supine position. The femoral artery was assessed from the level just distal to the inguinal ligament to the 2 cm distal to the junction of the common femoral vein and the greater saphenous vein. The collapsing of common and deep femoral veins was evaluated. The popliteal vein was assessed from the level of popliteal fossa up to the level of its trifurcation. The direct pressure with the use of transducer was applied in order to completely compress the vein. If the vein was compressed completely, then a DVT at this site was ruled out. The lack of possibility to completely compress the vein was treated as a positive test result (Fig. 1).

### Assessment of the right ventricle

**Fig. 2:**
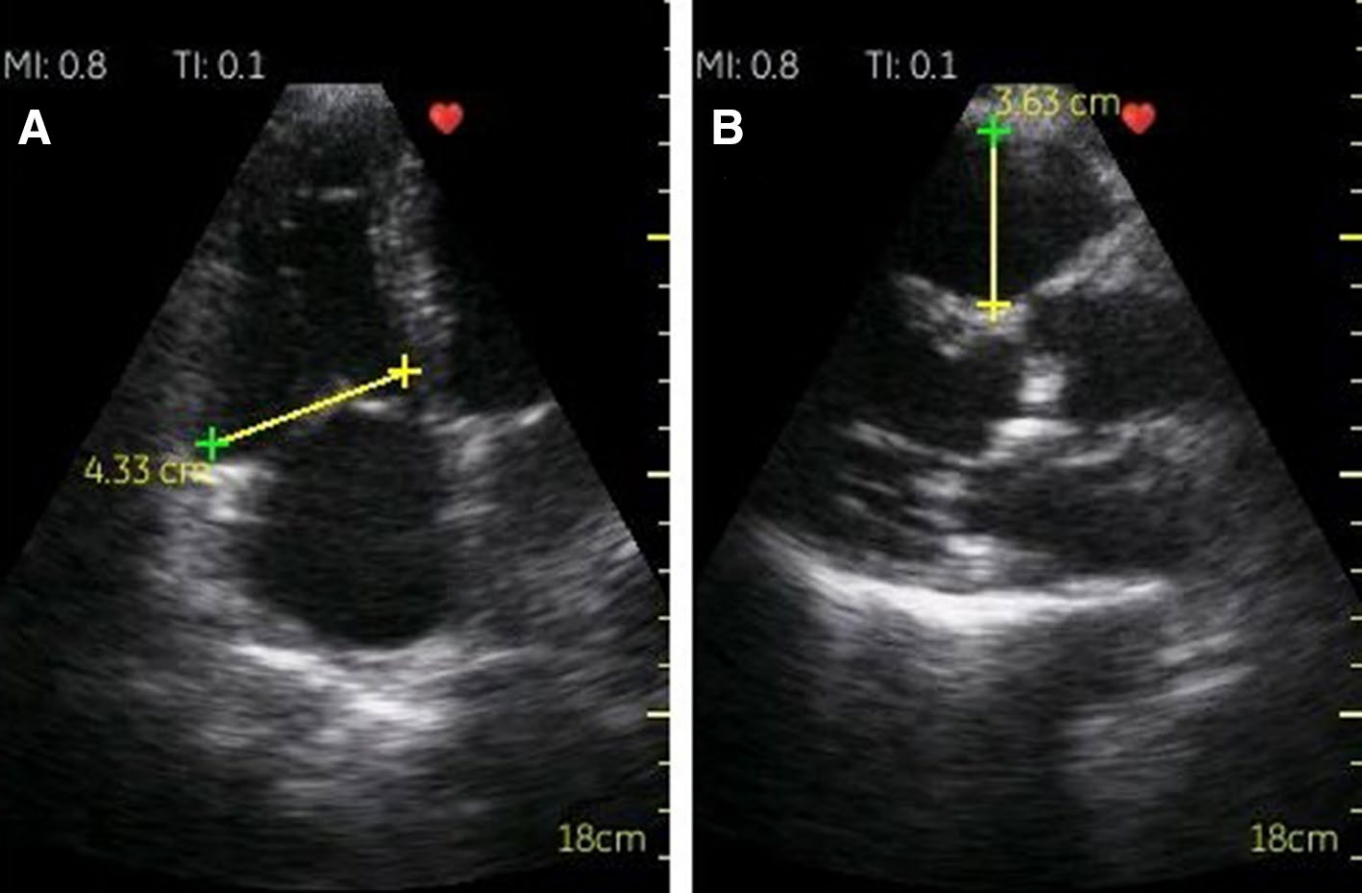
RV linear measurements: **a** RV focused 4-chamber apical view, basal dimension; **c** parasternal long-axis view, proximal RV outflow diameter

Two linear measurements of the right ventricle were performed: right ventricular basal diameter in the right ventricle-focused 4-chamber apical view and proximal right ventricle outflow diameter measured in long axis parasternal view (Fig. 2). Right ventricle enlargement was defined as the right ventricular basal diameter > 41 mm and/or proximal right ventricle outflow diameter > 35 mm [11].

### Modified clinical prediction rules

The points “clinical signs of DVT” in the Wells rule and “pain on lower limb deep venous palpation and unilateral oedema” in the revised Geneva score were changed into ‘positive CUS result’. Additional points were also given, when the RV enlargement was detected. Subsequently, we tested the hypothesis that incorporating the results of brief ultrasonographic assessment into the risk scale improves its diagnostic value. The score of this additional criterion was retrospectively determined on the basis of our population, to achieve the best ROC AUC. (Table 1).

### Diagnosis of pulmonary embolism

**Fig. 3:**
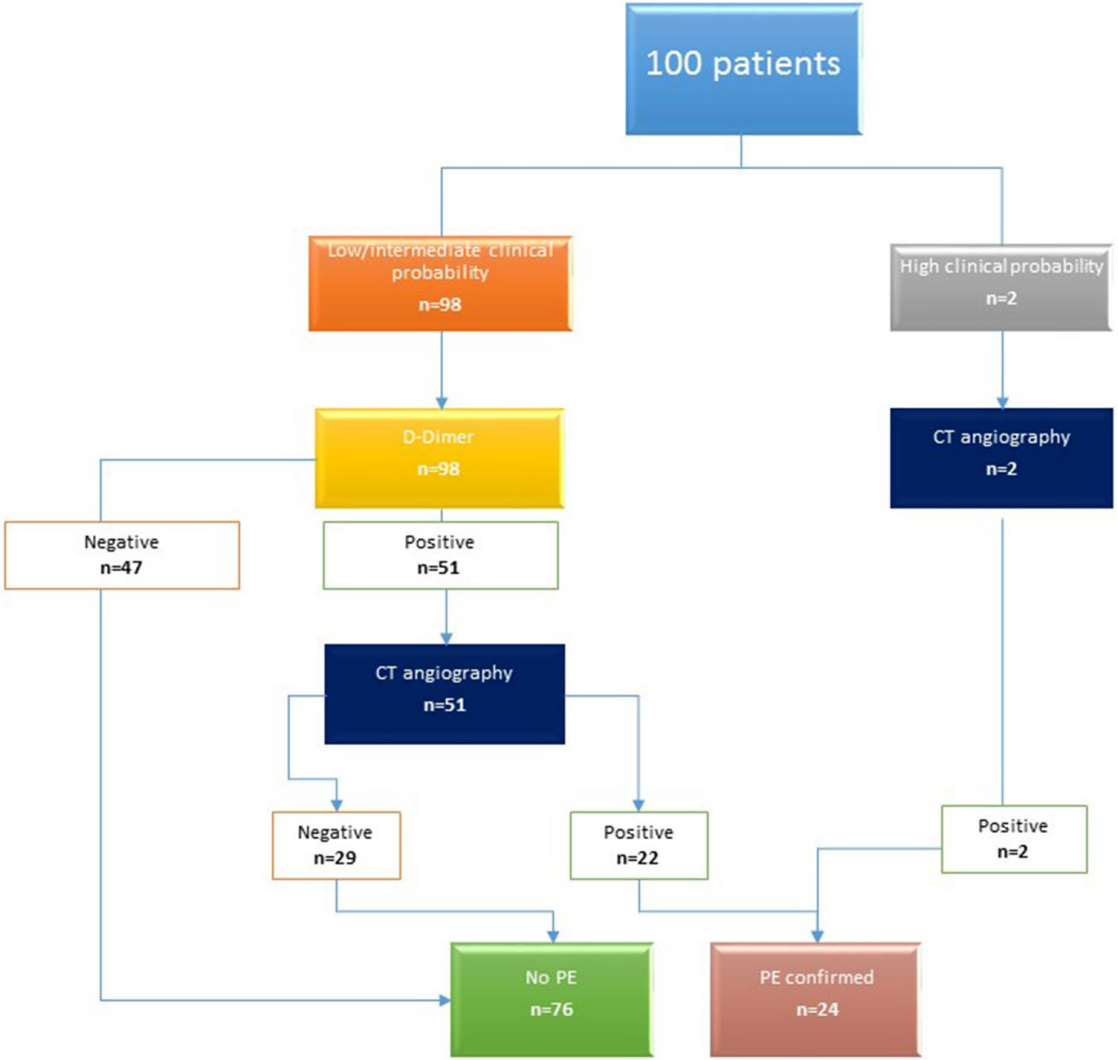
Diagram presenting the diagnostic path of patients included in the study

Final diagnosis was established in accordance with the algorithms recommended by the ESC guidelines, on the basis of clinical gold-standard including all necessary examinations [1]. Two patients with the high clinical probability of PE (according to the Wells score and revised Geneva score) immediately underwent CT-angiography. The remaining 98 patients with low or intermediate clinical probability had d-dimer plasma level initially assessed. In 47 patients with normal d-dimer plasma level (cut-off value: 500 µg/L) the pulmonary embolism was ruled out. In the remaining 51 patients CT angiography was performed (Fig. 3).

### Statistical analysis

Continuous and categorical variables are expressed as mean ± SD and as percentages (%), respectively. To assess the diagnostic value of different tests the ROC analysis on the basis of DeLong at al. methodology was performed. Sensitivity, specificity and overall diagnostic accuracy were compared with the use of N-1 Chi square test. A difference was considered statistically significant when p < 0.05. Calculations were performed with the use of MedCalc Software version 12.2.1.0.

## Results

### The assessment by recommended clinical prediction rules

**Fig. 4:**
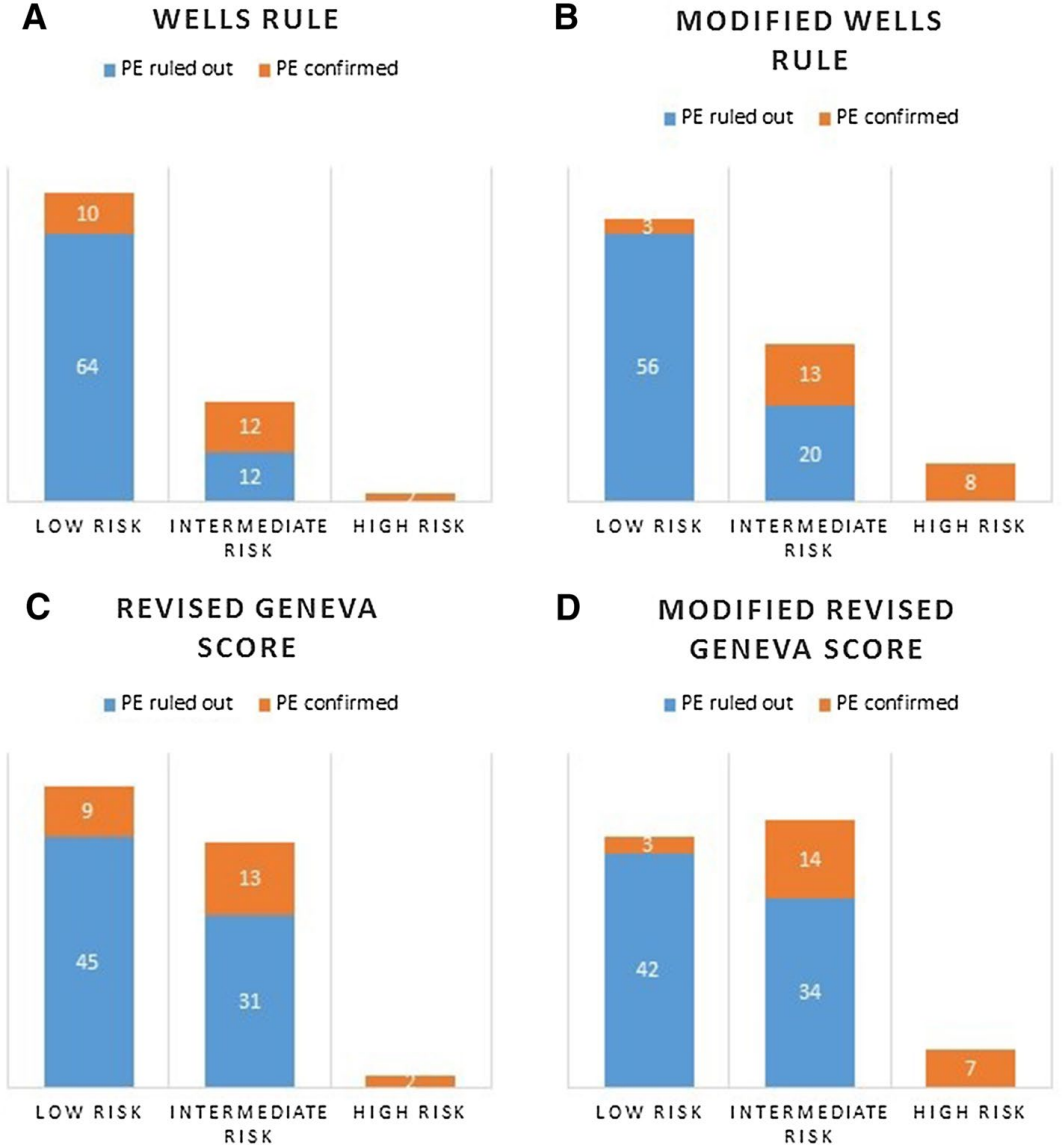
Clinical risk of PE estimated on the basis of three category Wells rule (Panel a) and revised Geneva score (Panel **c**); for modified Wells rule (Panel **b**) the following threshold were established: low 0–1, intermediate 2–6, high ≥ 7; for modified revised Geneva score (Panel **d**) the following threshold were established: low 0–3, intermediate 4–10, high ≥ 11

**Fig. 5:**
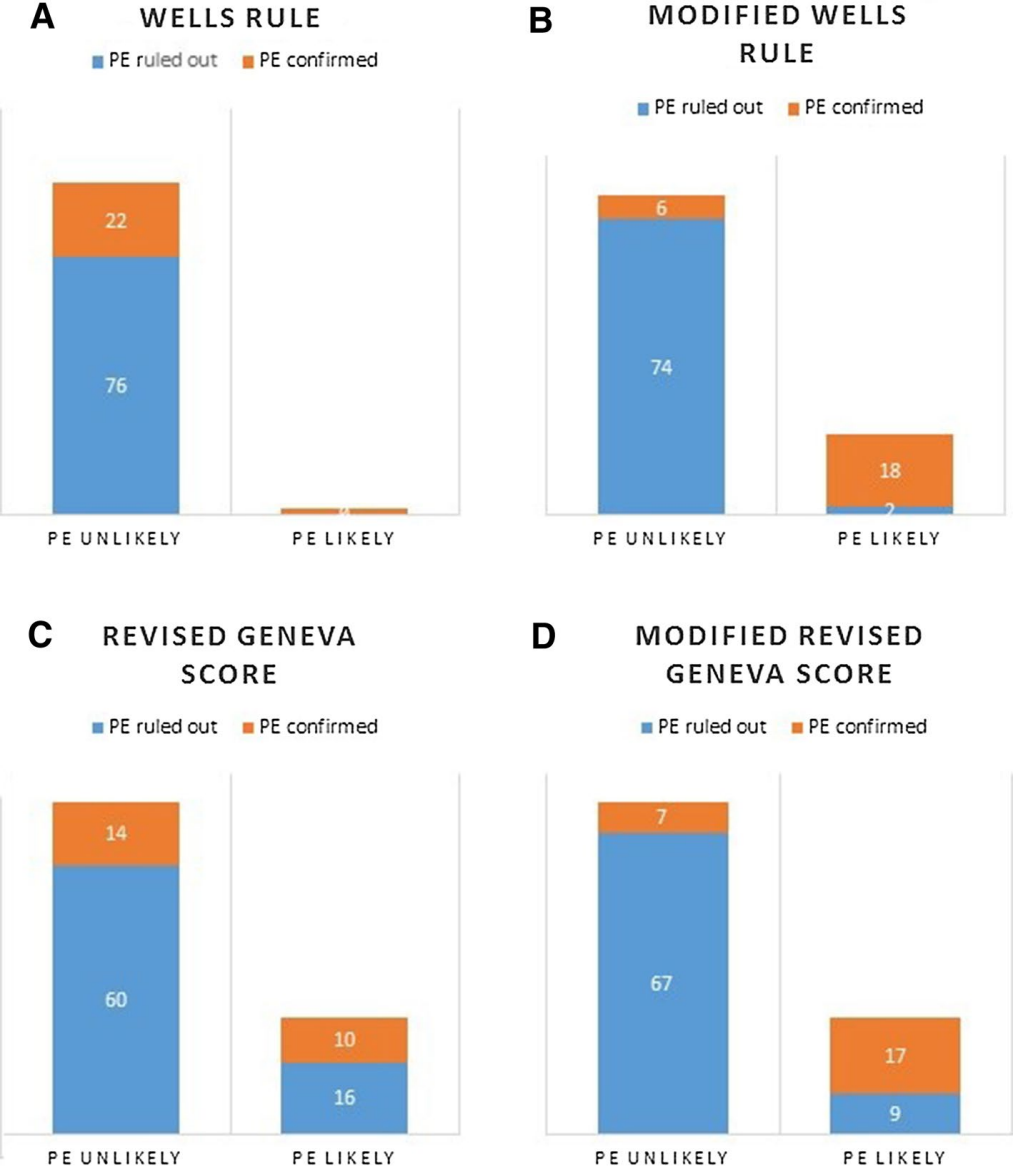
Clinical risk of PE estimated on the basis of two category Wells rule (Panel **a**) and revised Geneva score (Panel **c**); for modified Wells rule (Panel **b**) the following threshold were established: PE unlikely 0–3,5, PE likely ≥ 4; for modified revised Geneva score (Panel **d**) the following threshold were established: PE unlikely 0–6,5, PE likely ≥ 7

Pulmonary embolism was eventually confirmed by contrast-enhanced chest computed tomography in 24 patients. The patients’ characteristic is presented in Table 1. None of the patients had been given vasopressors on admission. In one patient the systolic blood pressure was < 90 mmHg. 17 patients had the history of chronic lung disease, in two patients atrial septal defect was present, three patients had the tricuspid regurgitation diagnosed. Among final diagnoses other than pulmonary embolism coronary artery disease (25 patients,) chronic heart failure exacerbation (22 patients), pneumonia (11 patients), heart rhythm disorders (seven patients), valve disease (six patients) were most often detected. According to the three-category Wells rule the clinical risk of PE was estimated as low in 74 patients, among which ten were eventually diagnosed with PE, as intermediate in 24 patients (12 cases of confirmed PE), as high in two patients (PE confirmed). In compliance with revised Geneva score, 54 patients had low clinical risk of PE (in nine patients PE was confirmed), 44 patients-intermediate (13 cases of PE); two patients-high (PE confirmed) (Figs. 4 and 5). The diagnostic accuracy of Wells rule and revised Geneva score based on our study population is presented in Table 2.

**Table 2:** The diagnostic accuracy of Wells rule, revised Geneva score, modified Wells rule and modified revised Geneva score (supplemented with the PSID test results) based on the study population

	Sensitivity (%)	Specificity (%)	PPV (%) (95% CI)	NPV (%) (95% CI)	Overall diagnostic accuracy (%)	ROC AUC (95% CI, p)
Wells rule ≥ 2^a^	66.7 (44.7–84.4)	85.5 (75.6–92.5)	59 (39–78)	89 (80–95)	81	0.776 (0.681 to 0.853, p < 0.0001)
Revised Geneva score ≥ 4^a^	62.5 (40.6–81.2)	57.9 (46.0–69.1)	35 (20–54)	82 (70–90)	59	0.664 (0.563 to 0.756, p = 0.0104)
Modified Wells rule ≥ 5^a^	70.8 (48.9–87.4)	98.7 (92.9–100.0)	94 (73–100)	92 (83–97)	93	0.914 (0.841 to 0.961, p < 0.0047)
Modified revised Geneva score ≥ 7^a^	75.0 (53.3–90.2)	88.2 (78.7–94.4)	67 (46–84)	92 (83–97)	94	0.877 (0.796 to 0.934, p < 0.0001)

^a^Thresholds: Wells rule ≥ 2, revised Geneve score ≥ 4, modified Wells rule ≥ 5, modified revised Geneve score ≥ 7; determined from the ROC curves

*ROC AUC* area under receiver operating characteristic curve, *NPV* negative predictive value, *PPV* positive predictive value

### Scanning with the use of PSID

The mean time of scanning with the use of PSID was 4.9 ± 0.8 (95% CI 4.7–5.0) min and was universally accepted by patients. One patient was excluded from the analysis, as the unilateral visualisation of popliteal vessels with the use of PSID was impossible. In two patients with the history of thoracotomy obtaining parasternal view proved impossible and RV size was determined in apical view exclusively. Fifteen patients had the deep vein thrombosis (five cases proximal) detected in compression ultrasound test, whereas RV enlargement was observed in 59 patients. Table 3 presents the diagnostic accuracy of the PSID scanning for identification of patients with PE calculated for various criteria of test positivity.

**Table 3:** The diagnostic accuracy of the PSID scanning for identification of patients with PE

	Sensitivity (95% CI)	Specificity (95% CI)	PPV (95% CI)	NPV (95% CI)	ROC AUC (95% CI, p)
Positive CUS	54% (33–74)	97% (91–100)	87% (58–99)	87% (78–93)	0.758 (0.662 to 0.838, p < 0.0001)
Proximal DVT diagnosed in CUS	21% (7–42)	100% (95–100)	100% (48–100)	80% (71–88)	0.604 (0.501 to 0.701, p = 0.0139)
RV enlargement	92% (73–99)	51% (40–63)	37% (25–51)	95% (84–99)	0.715 (0.616 to 0.801, p < 0.0001)
RV enlargement (basal 4CH) 2-Point scoring (42–47; > 47)	92% (73–99)	51% (40–63)	37% (25–51)	95% (84–99)	0.746 (0.649 to 0.828, p < 0.0001)
CUS and RV enlargement	54% (33–74)	100% (95–100)	100% (74–100)	87% (79–94)	0.771 (0.676 to 0.849, p < 0.0001)
CUS or RV enlargement	92% (73–99)	49% (37–60)	36% (24–50)	95% (83–99)	0.702 (0.602 to 0.789, p < 0.0001)

*ROC AUC* area under receiver operating characteristic curve, *CUS* compression ultrasound test, *DVT* deep vein thrombosis, *NPV* negative predictive value, *PPV* positive predictive value, *RV* right ventricle

### Modified clinical prediction rules

**Fig. 6:**
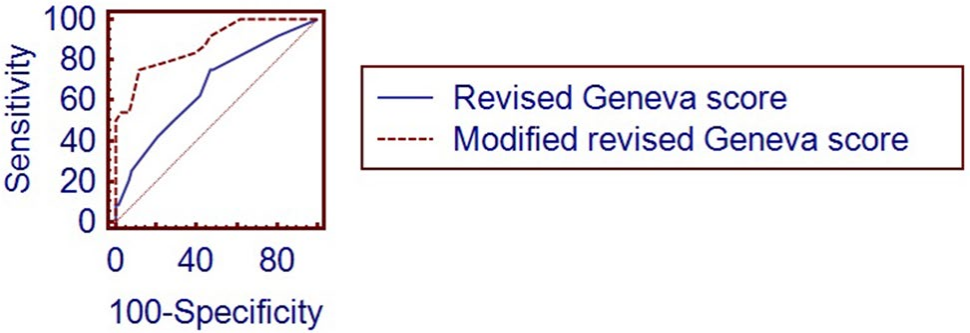
ROC curves comparison between revised Geneva score and modified revised Geneva score with additional criteria of CUS and RV enlargement

**Fig. 7:**
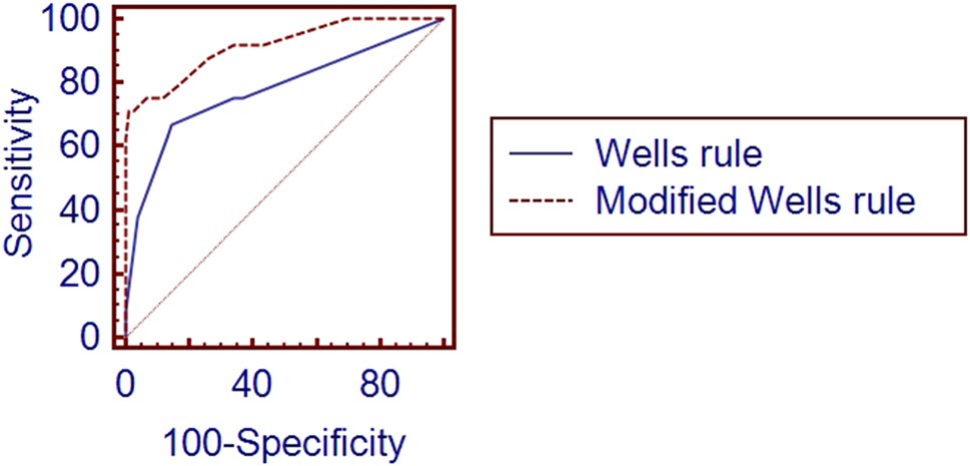
ROC curves comparison between Wells rule and modified Wells rule with additional criteria of CUS and RV enlargement

On the basis of our study population we have established that in case of positive lower extremity ultrasound the best ROC AUC were achieved for the values 4 points for Wells score and 9 points for revised Geneva score. For the RV enlargement we tested 1-point and 2-points scoring. The highest ROC AUC was obtained with two grade model: 1 point was added, if the basal diameter measured in the four chamber view was within the range of 42–47 mm, 2 points were given when this diameter exceeded 47 mm. Supplementing the revised Geneva score with additional criteria of positive CUS test and RV enlargement resulted in significant improvement of diagnostic accuracy of this score- difference between areas 0.212 (95% Cl 0.100–0.325, p < 0.0001), as presented on the graph. The overall diagnostic accuracy improved from 59 to 94% (p = 0.02). Similar modification of Wells score increased ROC AUC by 0.138 (95% CI 0.0429–0.223, p = 0.0045), the overall diagnostic accuracy from 81 to 93% (p = 0,012). Modification of both scales resulted in statistically significant improvement of specificity but not sensitivity. (Table 2; Figs. 6 and 7).

## Discussion

To the best of our knowledge our study is the first to report the diagnostic potential of brief scanning with the use of PSID in the initial assessment of patients with suspected pulmonary embolism. The main findings can be summarized as follows: (i) expanding the initial patient assessment in the ER with the elements of ultrasonographic imaging did not excessively prolong physical examination and was universally accepted by the patients (ii) simultaneous RV enlargement and positive CUS result identified with the use of PSID has a very high positive predictive value for PE (iii) the risk of PE assessed as low in accordance with the Wells and Geneva modified scores does not rule out the possibility of PE diagnosis (iv) the diagnostic value of the Well’s rule and the revised Geneva score can be significantly improved by implementing the results of PSID examination in the clinical prediction rules criteria.

Prompt diagnosis of pulmonary embolism still poses a challenge to clinicians, in spite of all the available modern diagnostic procedures. Based on the autopsy findings, diagnosis of PE is missed in up to 30–50% of patients [12]. 2/3 of deaths associated with PE occurs within the first hours from the symptoms manifestation [13]. For this reason any improvement in diagnostic accuracy and hastening of the whole diagnostic process is essential.PE and deep venous thrombosis (DVT) are considered as a continuum of the same clinical entity, namely venous thromboembolism. Even up to 90% of pulmonary emboli may arise from lower limbs or pelvis deep venous thrombosis [14]. The application of compression ultrasound in detection of the deep vein thrombosis in patients with suspected PE has been previously proposed and mentioned in recent guidelines as a method of reducing the number of CT angiography in appropriate patients. An early Dutch study based on the patients with suspected PE indicated that the use of compression US reduced the need for other imaging by 22%, at the expense of 2–4% of patients being unnecessarily treated for venous thromboembolism [15]. Data elicited from the metaanalysis indicate that CUS sensitivity ranged from 23 to 58%, while its specificity ranged from 89 to 99% [16]. Additionally CUS is commonly accepted as a non-invasive diagnostic modality, which may prove particularly relevant in patients with relative contraindications to CT, such as chronic kidney disease and creatinine clearance below 30 mL/min, allergy to iodinated contrast dye, pregnant women or younger patients, in which the reduction of irradiation is desirable.

The argument of limited cost-effectiveness was raised against the implementation of CUS in routine diagnostic process as it would require patient transportation to the ultrasound examination lab, specialized workforce and equipment. Adversely, according to ESC, examination with the use of PSID should not be treated as a separate procedure but rather as an augmentation of physical examination. PSID examination can be performed at the point of care. In contemporary clinical reality in which the concept of FOCUS is increasingly recognized and basics of ultrasonographic examination are becoming a vital part of numerous medical professionals’ training, one can expect that such diagnostic approach may not require additional personnel apart from the attending physician.

In agreement with the current clinical guidelines we have assumed in our group positive proximal CUS result would allow for instantaneous (on the level of ER examination) identification of PE in 5 (5%) patients. Among them in four patients with either low or intermediate clinical risk as estimated by means of Wells and revised Geneva scale PSID examination would help to avoid a prolonged wait for d-dimer test results and subsequent CT. In one patient with high clinical risk, immediate PE diagnosis would eliminate the need for CT scanning along with the potentially dangerous need for transportation. What is more, a DVT detected in CUS (also distal) accompanied by the RV enlargement in 100% of cases was related with the presence of thrombi in pulmonary circulation. One may hypothesize that also in the group of patients with the confirmed distal DVT, an additional screening for the RV enlargement could eliminate the need for blood testing/CT.

Although, in accordance with the guidelines, echocardiography does not play an essential role in the diagnostic process of PE, it can undoubtedly prove useful as a method of treatment progress assessment. Furthermore, it was previously confirmed that focused echocardiographic assessment, as a part of multiorgan bedside ultrasonography can improve clinical evaluation of patients with suspected PE prior to definitive imaging [17]. Pathologies described by the incorrect values of the ratio of RV to left ventricular end-diastolic diameter; RV systolic pressure, tricuspid annular plane systolic excursion and inferior vena cava collapsibility were confirmed to be related with the increased mortality during the course of acute PE [18]. Parameters related with RV dysfunction proved to have relatively high specificity while being burdened with low sensitivity [19]. In the study conducted by Kuznicka et al. [20] aimed at the assessment of the frequency of pathological findings in echocardiographic examination in patients with the confirmed PE, RV enlargement was relatively common, particularly in the high-risk patients. However, incorrect ventricular ratio criterion was not fulfilled in all such cases. Apart from that, RV enlargement as a quantitative parameter is in our opinion easier to objectively assess than qualitative criteria such as free wall hypokinesis or paradox movement of intraventricular septum. Due to the above mentioned rationale RV enlargement was chosen for the purpose of RV function evaluation.

RV dilatation as diagnosed during echocardiographic examination in patients with pulmonary embolism has been previously demonstrated to be related with the permanent RV dysfunction, RV failure, recurrent pulmonary embolism and death [19–22]. Although evaluating RV size and systolic function is not sufficient to make a direct diagnosis of PE it could provide an additionally valuable evidence in some patients. Should the dilated RV be detected during the bedside echocardiographic examination in a high-risk PE patient, the proper treatment introduction should be hastened with the improvement of morbidity and mortality [13].

In our study population, RV enlargement was a relatively common finding. Although such diagnosis may be associated with worse prognosis in patient with the suspected PE, it is important to point out that the majority of causes of RV enlargement in the study group was not related with PE. Thus, RV enlargement alone should not alter the diagnostic process and trigger prompt CT-scan. Nevertheless, the discussed parameter is useful as one of the factors reflected in risk scale.

It was previously established that trained emergency physician was able to perform a reliable evaluation of the RV dysfunction during a bedside examination [13, 23–29]. The efficacy of PSID screening in the assessment of the RV dilatation was also confirmed [30–32]. In our study population the prevalence of RV enlargement in PE patients was very high; only one patient with diagnosed PE could not be diagnosed with RV pathology.

Nazerian et al. [33] presented an appealing approach, in which they proved that enhancing the Wells rule with lung and lower limb venous ultrasound improved the diagnostic value of this scale. However, their approach still involved patient transportation, the use of high-end equipment and required involvement of a specialist in ultrasonography. One of the most highly regarded advantages of clinical prediction rules is the easiness of their application and the immediate result. PSID examination shares these features as it can be performed at any point of care, including the ER. Furthermore PSIDs can be operated by less experienced medical professionals who after the completion of the short training should be able to perform a reliable, specifically-aimed ultrasographic screening [8–10, 34–36]. Importantly, according to the ESC guidelines PSID screening should be integrated into the routine physical examination rather than treated as a separate diagnostic procedure.

Latest and most advanced PSIDs are equipped with a dual-probe, which shares the advantages of a linear and sector probe in one ultraportable tool. Older generations of PSID were not perfectly suited for the vascular imaging and significant shortcomings in this area were present. Clinicians had to overcome the obstacles of the minimal depth, image sector size and insufficient probe frequency [37]. PSID equipped with dual-probe appears to be capable of being successfully utilized in new clinical applications. Importantly, practicality of ultraportable ultrasound may suffer from limited imaging capabilities. Although in all 100 patients studied we were able to visualise RV at least partly, and sufficiently for measurements, this may not be possible in all-comers population in clinical setting. Lower extremity vein assessment may also exceed the capabilities of the device in some patients (in our study group ca. 1%). For this reason the supplementary role of PSID examination needs to be re-emphasized; final diagnosis should be obtained on the basis of the complete set of clinical data.

## Conclusion

Despite the well-established value of the PE clinical prediction rules, the diagnostic process of patients with suspected PE benefits from the addition of brief assessment with the use of PSID.

### Limitations

This is a single center study with relatively small study population. All of the examinations with the use of PSID were performed by the same cardiology resident, and for this reason the calculation of the inter-rater agreement index was not possible. RV analysis was limited to two linear measurements and its function was not evaluated. Compressive ultrasonography does not offer the possibility to diagnose pelvic deep venous thrombosis thus singular cases of proximal DVT could have remained undetected.
